# Editorial: Towards a better understanding of the physiology of the lymphatics

**DOI:** 10.1186/s41232-022-00200-2

**Published:** 2022-03-31

**Authors:** Masayuki Miyasaka

**Affiliations:** Osaka University Immunology Frontier Research Center, Suita, Japan

Among the many different COVID-19 vaccine platforms deployed so far, mRNA vaccines that deliver the genetic information of the SARS-CoV-2 spike protein into lymph nodes (LNs) have most successfully induced robust immune responses against SARS-CoV-2. In these vaccines, mRNA is encapsulated in cationic lipid nanoparticles (LNPs) that have the ability to enter into the lymphatic system through specific mechanisms. Consequently, after intramuscular injection, COVID-19 mRNA vaccines gain immediate access to the draining lymph nodes (LNs) via lymphatic vessels. The mRNA is then efficiently internalized and translated to the viral spike protein by antigen-presenting cells within the LNs, such as dendritic cells and macrophages; this process initiates both the innate and adaptive immune responses against SARS-CoV-2. These events occur first within the draining LNs of the injection site and subsequently in other lymphoid tissues, systematically generating the coordinated activation of T cells and B cells, which provides protection against this particular pathogen for a substantial length of time.

Lymphatic vessels are equipped with single-layered non-fenestrated endothelial cells. In the initial lymphatics, these endothelial cells are arranged in a gapped but overlapped manner in the absence of a complete basement membrane; this allows the directional entry of fluid and cells from the tissue into the lumen of the lymphatic vasculature under physiological conditions. Following the intramuscular injection of a COVID-19 mRNA vaccine, these absorptive processes are further enhanced, as cationic LNPs also function as an adjuvant, inducing inflammatory responses in the injection site and in the draining LNs. Consequently, neutrophils, monocytes, and dendritic cells are recruited to these sites, taking up the LNP-encapsulated mRNA vaccine and presenting it to T and B cells when they migrate into draining LNs. Collectively, these events promote protective immune responses against SARS-CoV-2.

Thus, lymphatics and their endothelial cells play an essential role in the modus operandi of mRNA vaccines, and a greater understanding of the structural and functional basis of how they coordinate local immune responses is critical to the development of improved vaccines for any pandemic threats.

In this thematic series review, we have invited world-leading researchers of lymphatic endothelial cells to share their insights and opinions. Masayuki Miyasaka has reviewed lymphatic endothelial cell heterogeneity. Yasuhiro Yoshimatsu and Tetsuro Watanabe have reviewed the roles of TGFβ signaling in the endothelial–mesenchymal transition in various physiological and pathological states. Fumiko Ito and others have also discussed the role of TGFβ signaling in the lymphatic metastasis of malignant tumors. Finally, Ryota Hokari and Akira Tomioka have reviewed the role of lymphatics in inflammatory bowel disease.

I would like to express my gratitude to the contributors of this special issue for sharing their time and expertise. I sincerely hope that these review articles will increase the depth of knowledge and extend our insight into the pathophysiology of the lymphatics and their endothelial cells.

February 18, 2022

Guest Editor

Masayuki Miyasaka

Osaka University Immunology Frontier Research Center

masayukimiyasaka@ifrec.osaka-u.ac.jp

